# Rapid sex determination of a wild passerine species using loop‐mediated isothermal amplification (LAMP)

**DOI:** 10.1002/ece3.5168

**Published:** 2019-04-15

**Authors:** Hanna R. Koch, Elke Blohm‐Sievers, Miriam Liedvogel

**Affiliations:** ^1^ Max Planck Research Group Behavioral Genomics Max Planck Institute for Evolutionary Biology Plön Germany

**Keywords:** blackcap, CHD‐W, CHD‐Z, Loop‐mediated isothermal amplification, molecular sexing, passerine, sex determination, *Sylvia atricapilla*

## Abstract

Many bird species are sexually monomorphic and cannot be sexed based on phenotypic traits. Rapid sex determination is often a necessary component of avian studies focusing on behavior, ecology, evolution, and conservation. While PCR‐based methods are the most common technique for molecularly sexing birds in the laboratory, a simpler, faster, and cheaper method has emerged, which can be used in the laboratory, but importantly also in the field. Herein, we used loop‐mediated isothermal amplification (LAMP) for rapid sex determination of blood samples from juvenile European blackcaps, *Sylvia atricapilla*, sampled in the wild. We designed LAMP primers unique to *S. atricapilla* based on the sex chromosome‐specific gene, chromo‐helicase‐DNA‐binding protein (CHD), optimized the primers for laboratory and field application, and then used them to test a subset of wild‐caught juvenile blackcaps of unknown gender at the time of capture. Sex determination results were fast and accurate. The advantages of this technique are that it allows researchers to identify the sex of individual birds within hours of sampling and eliminates the need for direct access to a laboratory if implemented at a remote field site. This work adds to the increasing list of available LAMP primers for different bird species and is a new addition within the Passeriformes order.

## 1 INTRODUCTION

With more than half of all avian species being monomorphic, determining the sex of a bird based on external morphology or obvious phenotypic differences, such as plumage color or patterns, becomes impossible. Even for sexually dimorphic species, sex determination during the life stages before adulthood can be extremely difficult. However, quickly and confidently identifying the sex of a [young] bird may be important for a variety of reasons, whether for conservation (Lambertucci, Carrete, Speziale, Hiraldo, & Donazar, 2013), wildlife management (Chan et al., 2012; Geigenfeind, Vanrompay, & Haag‐Wackernagel, 2012), or research purposes (Ito, Sudo‐Yamaji, Abe, Murase, & Tsubota, 2003; Lee et al., 2010; Vucicevic et al., 2013).

In many species, the sex of an individual is genetically determined by genes on one of two sex chromosomes, with one sex being homogametic and the other heterogametic. In birds, females are heterogametic (ZW) and males are homogametic (ZZ; Harris & Walters, 1982). Once it was discovered that the W chromosome contained the chromo‐helicase‐DNA‐binding gene (CHD gene; Griffiths & Tiwari, 1995), that a closely related copy occurred on the Z chromosome (Griffiths & Korn, 1997), and that both genes (CHD‐W/CHD‐Z) were highly conserved (Ellegren, 1996; Ellegren & Sheldon, 1997), they were quickly used as universal molecular markers for sexing birds (Griffiths, Double, Orr, & Dawson, 1998; Lee et al., 2010; Vucicevic et al., 2013).

A variety of molecular methods have been developed for using CHD in sex determination, including restriction fragment length polymorphism (RFLP), and multiple PCR‐based methods, with the latter being more common (Ellegren, 1996; Fridolfsson & Ellegren, 1999; Griffiths et al., 1998; Ito et al., 2003; Kahn, St John, & Quinn, 1998; Morinha, Cabral, & Bastos, 2012; Vucicevic et al., 2013). While PCR has become the standard for amplification of specific DNA sequences, this technique requires high‐precision instruments for amplification, such as a thermal cycler, and/or an extended protocol, gel electrophoresis for example, for visualization of the amplified product. This can lead to needing more time, money, and supplies for carrying out DNA amplification. However, relatively recently a new amplification technique has been developed, called loop‐mediated isothermal amplification (LAMP), which amplifies DNA with high specificity, efficiency, and speed under isothermal conditions (Notomi et al., 2000), and without the need for direct access to a laboratory (Lee, 2017).

**Figure 1:**
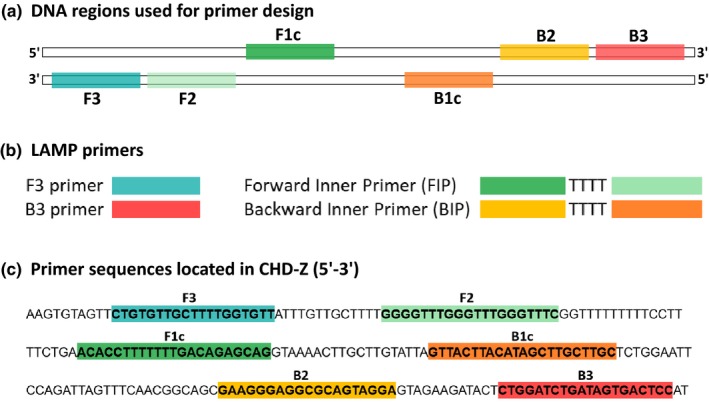
Schematic illustration of LAMP primer design for CHD‐Z. (a) Four different regions (forward: F2, F3; backward: B2, B3) and two complementary regions (forward: F1c; backward: B1c) are located on target DNA. (b) LAMP primers: two outer primers (forward: F3; backward: B3) and two inner primer pairs (forward: FIP; backward: BIP) are used in each LAMP reaction. The FIP (BIP) primer is generated by connecting the F1c (B1c) sequence and F2 (B2) sequence with a T‐linker. (c) Primer sequences located in CHD‐Z (5’‐3’) showing the six different target regions (forward: F3, F2, F1c; backward: B3, B2, B1c). Primer sequences for B3, F1c, and B2 are ordered as the reverse complements to the sequences above (see Tomita et al., 2008 and Centeno‐Cuadros et al., 2017; see also Table 1). Illustration is adapted from Centeno‐Cuadros et al. (2017)

Logistically, this new method offers a simpler protocol with the LAMP reaction requiring a single enzyme (DNA polymerase with strand displacement activity that can greatly amplify from a minimal number of DNA copies) and a single temperature, which removes the need for an expensive, high‐precision thermal cycler. Moreover, product detection can be achieved directly within the reaction tube by a diagnostic color change and fluorescence, eliminating the need for electrophoretic techniques and related equipment (Centeno‐Cuadros, Abbasi, & Nathan, 2017; Lee, 2017; Mori, Nagamine, Tomita, & Notomi, 2001). The principle of this method, based on autocycling strand displacement, is however more complex than traditional PCR and requires four specially designed primers that recognize six distinct regions on the target DNA (Figure 1). The process by which LAMP recognizes the target allows for amplification of the target sequence with high selectivity, and the final products are stem‐loop DNAs with several inverted repeats of the target in the same strand (for a detailed description of the LAMP mechanism, please see Notomi et al., 2000 and Tomita, Mori, Kanda, & Notomi, 2008).

Since its development, LAMP has been used widely across clinical research, medicine, microbiology, and parasitology, but less so within ecology and evolution. This method has also been used for sex determination in a number of organisms, including plants (Fujita et al., 2017; Hsu, Gwo, & Lin, 2012; Shiobara et al., 2011), fish (Hsu, Adiputra, Ohta, & Gwo, 2011), mammals (Khamlor et al., 2015; Nogami, Tsutsumi, Komuro, & Mukoyama, 2008), and birds (Centeno‐Cuadros et al., 2017; Centeno‐Cuadros, Tella, Delibes, Edelaar, & Carrete, 2018; Chan et al., 2012; Kim et al., 2015). Of the avian studies, only one of them has used LAMP as a field application (Centeno‐Cuadros et al., 2017; Lee, 2017). Herein, we followed Centeno‐Cuadros et al. (2017) for generating a LAMP‐based protocol to quickly sex juvenile blackcaps (*Sylvia atricapilla*, Order Passeriformes) in the laboratory or wild.

**Figure 2:**
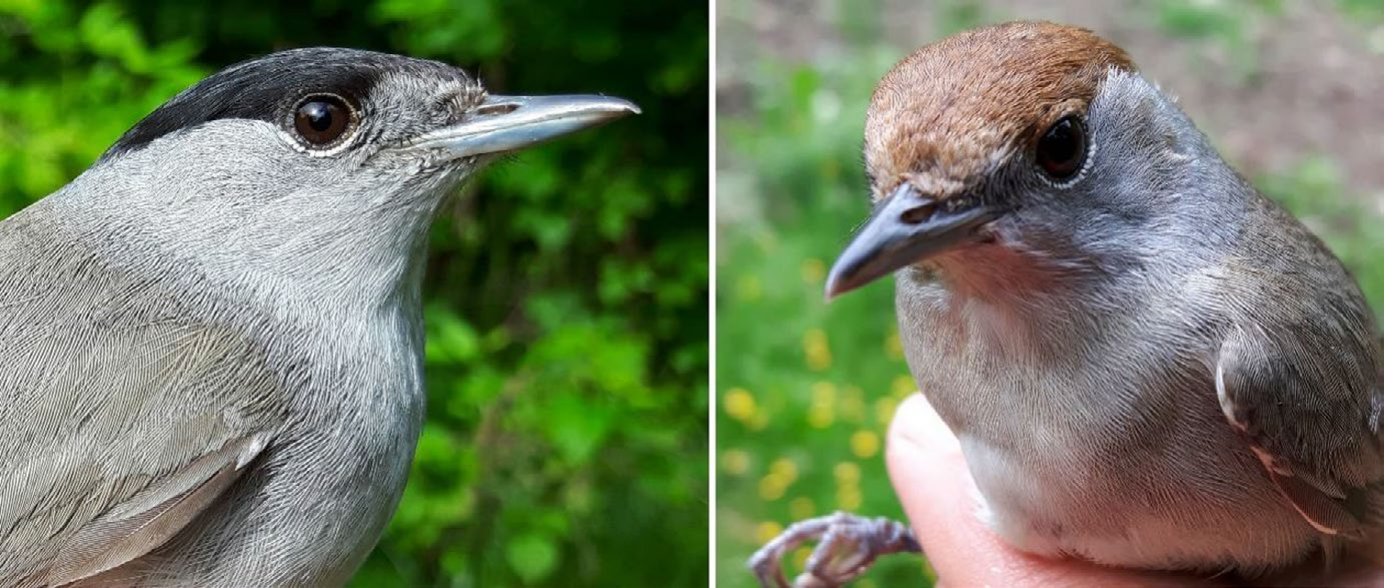
The European blackcap (*Sylvia atricapilla*). Adult male (left) and female (right) birds with characteristic black (m) and brown (f) “caps”

The blackcap is a common European passerine and serves as a model system for studying migration ecology as well as the underlying evolutionary genomic machinery controlling this complex behavioral phenomenon. Blackcaps exhibit the entire spectrum of migratory phenotypes, with variability in both migratory distance and orientation (Berthold, Helbig, Mohr, & Querner, 1992; Cramp, 1992; Helbig, 1991; Perez‐Tris, Bensch, Carbonell, Helbig, & Telleria, 2004). For example, blackcap populations may comprise short/medium/long‐distance migrants, partial migrants, or nonmigrants (i.e., they are resident populations; Pulido & Berthold, 2010). Regardless of migratory behavior, blackcaps after their first post‐juvenile molt are sexually dimorphic, where males have a black patch (“cap”) on top of their heads and females have a brown one (Figure 2). However, male and female juveniles of this species, which have not undergone their first molt, all have a brown cap and are indistinguishable based on plumage color patterns. Therefore, any study for which gender is an important factor, but utilizes wild‐caught juvenile blackcaps prior to their first autumn migration, will suffer a time lag between sampling and obtaining molecularly derived sex determination results. This time lag, coupled with the inability of researchers to make decisions in situ and in real time concerning individual gender, may slow progress considerably and involve logistical obstacles, such as, needing to keep juvenile birds in cages until their sex has been determined and/or requiring access to a nearby laboratory, which may or may not be possible given the sampling location and/or research affiliations. Having a rapid sex determination protocol that can be easily implemented in the field would remove some of the associated logistical limitations, as well as simplify and hasten sex determination of samples processed in the laboratory.

Herein, we used a subset of naïve, wild‐caught juvenile blackcaps, which were sampled from southern Spain, as part of a larger evolution and behavioral study on migratory behavior, to test the applicability of our optimized LAMP primer sets for sex determination in this species. For the behavioral study, the target gender was male as it is the homogametic sex in birds (ZZ), and thus, whole‐genome sequencing (a component of the behavioral study) results in equal coverage of the Z chromosome and autosomes. The behavioral study is an example of a study that would benefit greatly from a LAMP‐based sexing protocol, where in situ sex determination would lead to rapid identification and collection of male samples, along with instant identification and subsequent release of female birds, thereby reducing stress on them.

Once birds were captured in the field, a blood sample was taken from each individual and samples were then transported back to a laboratory for sex determination and further analyses. We selected a random subset of samples to be tested using a standard PCR‐based protocol (Fridolfsson & Ellegren, 1999; Griffiths et al., 1998) and LAMP‐based protocol to confirm gender and test reliable applicability of the LAMP method in this setting. Our optimized LAMP‐based protocol proved to be just as accurate, but much more time efficient compared to the PCR‐based protocol.

## 2 MATERIALS AND METHODS

### 2.1 Sample collection

The collection of birds was designed in a way to support the needs of the larger behavioral study, and the specifics of collection, for example, site, date, and migratory phenotype, are not necessarily relevant for the present study. In summary, 67 birds were collected in August 2017 from two different field sites in southern Spain. Birds were captured using mist nets and tape luring, using audio recordings of the male blackcap territorial song. Blood samples (ca. 50 µl) were taken, using a capillary tube, from the brachial vein and stored in 500 µl SET buffer (0.015 M NaCl, 0.05 M Tris, 0.001 M ethylenediaminetetraacetic acid, pH 8.0) and stored in a −20°C freezer until tested. We chose blood over feathers, for example, because bird blood is a better tissue for obtaining high amounts of DNA; nucleated erythrocytes provide much more DNA compared to relatively low amounts of DNA recovered from feather samples (Harvey, Bonter, Stenzler, & Lovette, 2006). Eighteen samples with a roughly equal representation of males and females were randomly chosen to be tested, using both PCR and LAMP protocols in the laboratory.

### 2.2 DNA extraction optimization

Prior to testing the samples with LAMP, we optimized DNA extraction using blood samples from adult birds of known gender (positive controls; data not included). Using no more than half of the sample volume of blood in SET buffer (~250 µl), DNA was extracted using a simple and fast HotSHOT DNA extraction method (Truett et al., 2000), which uses sodium hydroxide (NaOH), requires less than 45 min, and can be carried out using a single thermoblock set to 95°C. For a detailed protocol, please see Truett et al., 2000, as we followed it directly. Optimizing incubation timing (10 min, 30 min, 1 hr) resulted in 30 min incubation duration working best. We optimized starting DNA amount by testing a range of concentrations (1.5–3.5 ng/µl, 15–45 ng/µl and ~400 ng/µl) and found <5 ng/µl to be optimal. LAMP is known to be highly sensitive and able to amplify only a few copies of DNA. DNA yield was quantified using a NanoDrop™ spectrophotometer.

We also optimized the DNA extraction protocol to be used as a “quick and dirty” method for field application, where access to a nucleic acid quantification machine, for example, NanoDrop™, is unlikely. While a 50 µl blood sample is recommended, variability/difficulty in handling live animals (i.e., sampling error) may lead to more or less than that. Therefore, blood samples should be binned into three different categories, based on the relative color of the sample. More concentrated samples will appear dark red while less concentrated samples will appear diluted and lighter in color. We applied this system to our known control samples and designated them as light, medium, or dark red (with two replicates each), carried out the HotSHOT DNA extraction protocol, quantified DNA yield using a NanoDrop™, performed 1:1, 1:5, 1:10, and 1:100 dilutions to determine final DNA concentrations and which would be optimal for the subsequent LAMP protocol. Too much or too little DNA will lead to failed LAMP reactions. We found light and medium samples (30–50 µl blood + 500 µl SET buffer) yielded ~130–150 ng/µl of isolated DNA; therefore, 1:30 dilutions would be necessary. Alternatively, dark samples (>50 µl blood + 500 µl SET buffer) yielded ~450–550 ng/µl of isolated DNA, so 1:100 dilutions would be sufficient for more concentrated blood samples. While it is an imprecise method for estimating DNA concentration, we found it to work reliably across the tested samples in the laboratory, and therefore know it will also work robustly in the field.

### 2.3 LAMP primer design and optimization

Sex determination using LAMP requires two sets of primers and thus two reactions per sample, based on CHD‐Z and CHD‐W genes. CHD‐Z primers target the Z chromosome which is found in both males (ZZ) and females (WZ) and will therefore show a positive LAMP reaction for both sexes. A CHD‐Z reaction that produces a negative result indicates a problem, and a repeat test should be conducted. In this sense, CHD‐Z reactions can be considered positive controls of DNA quality. LAMP reactions targeting the W chromosome (CHD‐W gene) will only produce positive results for female samples and are therefore necessary to reliably distinguish males from females; negative LAMP reactions for CHD‐W indicate a male, but only if the accompanying Z reaction is positive.

Primer design and selection was done following Tomita et al. (2008) and using PRIMER EXPLORER V4 software (Eiken Chemical Co., Ltd., http://primerexplorer.jp/e/). For each target gene, CHD‐W and CHD‐Z, we designed a primer set consisting of four different primers: forward (F3), backward (B3), forward internal primer (FIP), and backward internal primer (BIP) (e.g., Figure 1; Table 1). To design CHD‐Z primers, we used the CHD‐Z gene sequence of an in‐house blackcap reference genome (male, ZZ; Delmore *et al.* under review) and the LAMP primer design software (PRIMER EXPLORER). Since our in‐house blackcap genome was male (ZZ), we could not use it to design primers for the CHD‐W gene. Instead, we used the LAMP primer design software to generate a consensus sequence for the CHD‐W gene based on partial gene sequences from three other avian species *Taeniopygia guttata* (zebra finch), *Gallus gallus* (chicken), and *Gyps fulvus* (Griffon vulture) (GenBank Accession numbers: AF006662.1, AF006660.1, EU430640.1, respectively). We used a feature implemented within the primer design software to flag and avoid polymorphic positions when generating species‐specific primers for the blackcap. Multiple primer sets were designed and ordered for each target gene (CHD‐W and CHD‐Z), with FIP/BIP synthesized using a HPSF (high purity salt free) method. Alternatively, they can be purified using HPLC (high‐performance liquid chromatography), as recommended by Tomita et al. (2008).

**Table 1:** LAMP primers, including sequences and optimal amplification conditions, designed to amplify species‐specific CHD‐W and CHD‐Z genes for *Sylvia atricapilla*

Target	Primer	Name	5′‐Sequence‐3′	Temperature (°C)/Time (min)
CHD‐Z	F3	Z‐F3	CTGTGTTGCTTTTGGTGTT	65°/80′
	B3	Z‐B3	GGAGTCACTATCAGATCCAG	
	FIP	Z‐FIP	CTGCTCTGTCAAAAAAAGGTGT**TTTT**GGGGTTTGGGTTTGGGTTTC	
	BIP	Z‐BIP	GTTACTTACATAGCTTGCTTGC**TTTT**TCCTACTGCGCCTCCCTTC	
CHD‐W	F3	W‐F3	ACTTAATCTGAAATTCCAGATCA	65°/60′
	B3	W‐B3	TCTGCATCGCTAAATCCTT	
	FIP	W‐FIP	AGTCACTATCAGATCCAGAATAT**TTTT**GCTTTAATGGAAGTGAAGGGA	
	BIP	W‐BIP	TCTCAGAAAGAAAACGACCA**TTTT**TATTTTCTCGAGGAATAGTTCGC	

Note

F/B3 = forward/backward external primer; F/BIP = forward/backward internal primer pair composed of F/B1c and F/B2, joined by a T‐linker (bold).

To optimize primers, we used the same set of blood samples of known gender that was used as positive controls for optimizing DNA extraction and a gradient thermal cycler to test a range of reaction temperatures (55, 57, 61, 63, 65, 67, 69°C) and reaction times (45, 60, 80, 90, 120 min) (data not shown). For determining optimal incubation time, we selected the shortest incubation time that yielded consistently accurate results. Any primer set (F3, B3, FIP, and BIP for CHD‐Z or CHD‐W) that yielded no amplification for any combination of temperature and time was discarded, and a new primer set was randomly selected and tested. Final primer sets, along with sequences and optimal amplification conditions, are described in Table 1.

### 2.4 Sex determination using LAMP

For setting up LAMP reactions, we used a protocol that was developed and tested in the laboratory but could be easily modified for application in the field. We followed Hamburger et al. (2013) in preparing a ready‐mix solution of reagents that would preserve enzyme activity while allowing us to work at room temperature with the possibility to store it for months without freezing. The ready‐mix solution included 1x enzyme buffer (10 mM Tris‐HCl, 3.5 mM MgCl_2_, 75 mM KCl, pH 7.5), 0.4 mM dNTPs, 1 M Betaine, 2% sucrose stabilizer solution (Centeno‐Cuadros et al., 2017), and 8U of Bst DNA polymerase (New England Biolabs), per reaction (Hamburger et al., 2013). We used Bst WarmStart 2.0 to eliminate the need for working on ice.

Two 25 µl LAMP reactions were set up per sample, one targeting CHD‐Z and the other CHD‐W. Each reaction tube received 14 µl of the reagents ready‐mix solution, 5 µl dd‐H_2_0, and 1 µl of each primer (F3/B3 final concentration 5 µM; FIP/BIP final concentration 40 µM). We received the synthesized primers as lyophilized, resuspended them using molecular grade H_2_O and stored them in the −20°C freezer until use. Finally, 2 µl of the template DNA (1:100 dilution or 1:30 dilution, depending on the initial blood concentration) was added to each reaction tube, mixed well, and then incubated in the same thermoblock that was utilized for DNA extractions, at 65°C for 60 min (CHD‐W reactions) or 80 min (CHD‐Z reactions; Table 1).

Detecting successful LAMP reactions was achieved in three ways: gel electrophoresis, staining, and fluorescence. LAMP amplicons were visualized by running a 2.5% agarose gel (100 V, 40 min), again keeping CHD‐W and CHD‐Z reactions separate for each sample. Then we added 5 µl of 1:50 diluted SYBR Green I Nucleic Acid Stain (Life Technologies) to each reaction tube. An immediate color change in the tube, from orange to yellow, indicates a positive LAMP reaction; the staining reagent reacts with magnesium pyrophosphate residuals created during the DNA synthesis in LAMP and turns bright yellow (Parida, Sannarangaiah, Dash, Rao, & Morita, 2008). A negative LAMP reaction will not produce a color change. The color change can also be easily visualized by irradiating the LAMP reactions with UV light (320 nm). Positive reactions will fluoresce (yellow), negative reactions will not.

### 2.5 Sex determination using PCR

To corroborate LAMP results, we tested the same 18 samples using a standard PCR‐based molecular sexing protocol and a single set of primers, P2 and P8, which are also designed from the CHD genes (for a detailed description of the protocol and primers, please see Griffiths et al., 1998). PCR amplification was carried out using a thermal cycler and lasted roughly two hours. Afterward, PCR products were visualized by running a 3% gel agarose electrophoresis (100 V, 2.5 hr). A single band (CHD‐Z) indicates a male, whereas the presence of a second band (CHD‐W) indicates a female. For this test, the number of bands serves as the diagnostic indicator, not fragment size.

## 3 RESULTS

### 3.1 LAMP optimization and reactions

**Figure 3:**
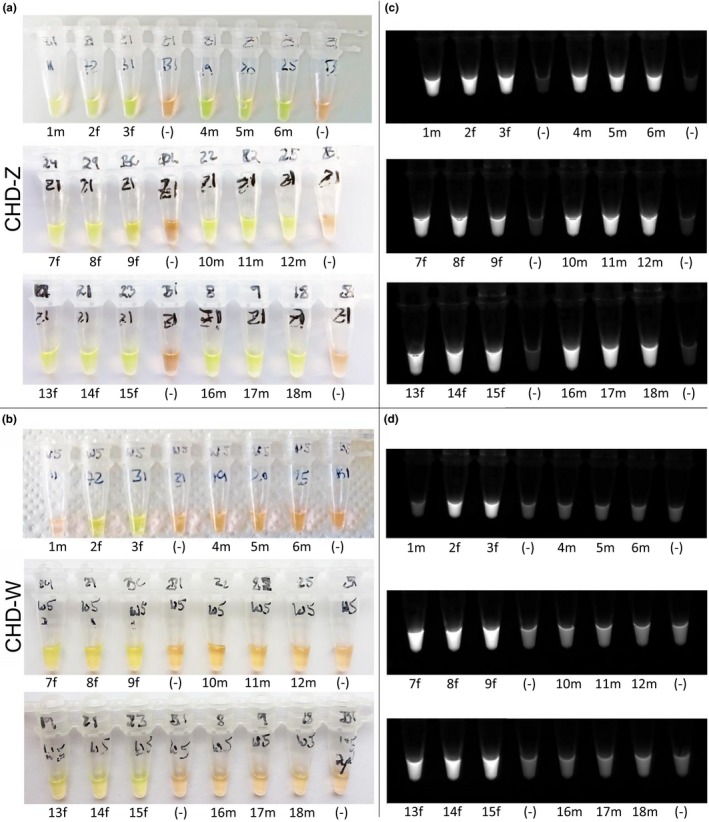
LAMP results for rapid sex determination of wild‐caught juvenile blackcaps, *S. atricapilla*. Two LAMP reactions were run for each sample (*n* = 18), using primer sets amplifying fragments of CHD‐Z (a, c) and CHD‐W (b, d) genes. (a–b) Positive LAMP reactions were visualized by an immediate color change, orange to yellow, after staining with SYBR Green I. Negative reactions remained orange. Across samples, eight were reliably identified as female (“f”, positive CHD‐Z and CHD‐W reactions; both yellow) and ten were male (“m”, positive CHD‐Z reactions (yellow) but negative CHD‐W reactions (orange)). Negative controls (−) were included to test for spurious amplification. (c–d) Positive LAMP reactions were confirmed by irradiating tubes with UV light and detecting fluorescence (here, color is inverted to see the contrast). Negative reactions did not fluoresce. The same samples, 2–3, 7–9, and 13–15 reliably revealed to be female (“f”) while the rest were male (“m”; 1, 4–6, 10–12, and 16–18)

After testing different combinations of incubation temperature and time, we found that males and females were clearly and consistently distinguished when LAMP reactions were run at 65°C for 60 min (CHD‐W primers) and 80 min (CHD‐Z primers; Table 1). Visualization of amplicons under ambient light conditions was immediate after staining with SYBR Green I and seeing the sample color change from orange to yellow for all 18 reactions targeting CHD‐Z (Figure 3a) but only for eight of the 18 reactions targeting CHD‐W (Figure 3b), indicating the presence of eight females and ten males. This result was confirmed after exposing the tubes to UV light and seeing fluorescence for all CHD‐Z reactions (Figure 3c), and the same eight CHD‐W reactions (samples 2–3, 7–9, and 13–15; Figure 3d).

### 3.2 *Comparison of LAMP *versus* PCR*

**Figure 4:**
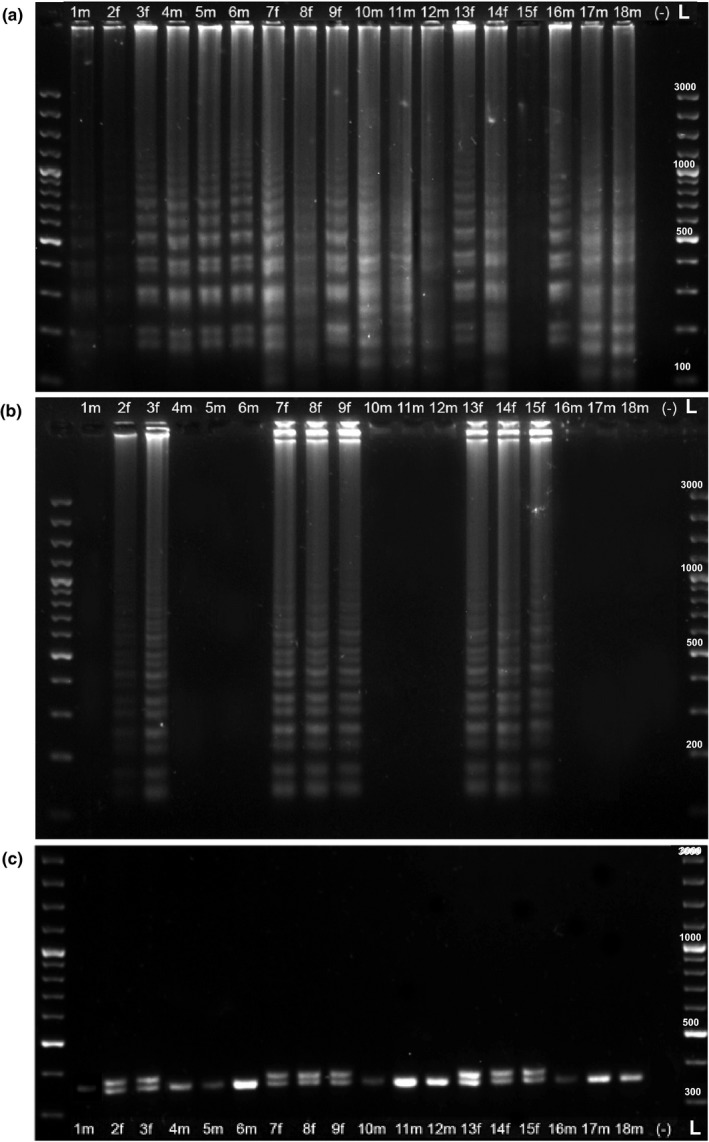
Agarose gel images of LAMP and PCR amplicons for 18 wild‐caught, juvenile blackcaps. (a–b) LAMP amplicons, for CHD‐Z (a) and CHD‐W (b) reactions, were run on a 2.5% agarose gel. Positive LAMP reactions produce a ladder‐like pattern on the gel, which can be seen for all 18 CHD‐Z reactions, but only for the CHD‐W reactions corresponding to samples 2–3, 7–9, and 13–15, indicating that they are female while the others are male. (c) PCR amplicons for each of the 18 reactions were run on a 3% agarose gel with the same DNA ladder (left and right). A single band indicates a male (“m”; samples 1, 4–6, 10–12, and 16–18); two bands indicate a female (“f”; samples 2–3, 7–9, and 13–15). (L) GeneRuler 100 bp Plus DNA Ladder. Negative controls (−) were included in both tests

We tested the same 18 samples using a standard molecular sexing protocol (PCR‐based) to confirm LAMP results and compare the efficiency of the protocols in the laboratory. We visualized amplified products from both protocols by running an agarose gel electrophoresis and found that results corroborated each other, with samples 2–3, 7–9, and 13–15 determined to be female while the rest were identified as male (Figure 4). For the LAMP reactions, CHD‐W and CHD‐Z products were run in parallel for each sample, and a ladder‐like pattern, indicating a positive LAMP reaction, was observed for all CHD‐Z reactions (Figure 4a), but only eight CHD‐W reactions (samples 2–3, 7–9, 13–15; Figure 4b). For the PCR amplicons, each sample was run in a single lane and results revealed the same pattern of sex determination, eight females (two bands) and ten males (1 band) (Figure 4c).

## 4 DISCUSSION

Herein, we designed species‐specific LAMP primers for rapid sex determination of wild‐caught juvenile blackcaps, *S. atricapilla*. We optimized the entire protocol, including DNA extraction and LAMP, in a way that would allow for fast and easy application in both the laboratory and a field setting. Consequently, one of our goals was to find one single incubation temperature that would work accurately for both CHD‐W and CHD‐Z primer sets so that separate reactions could be run simultaneously on the same thermoblock, requiring less equipment and time overall. We found that when incubated at 65°C, LAMP could clearly and reliably distinguish male and female samples, with only a 20‐min difference in incubation times for CHD‐W and CHD‐Z primers. Therefore, one set of reactions can be easily removed from the thermoblock without disrupting the other set, and processed separately, which is ideal when handling LAMP reactions to reduce the risk of cross‐contamination and false positives.

The most commonly reported drawback of LAMP is high rates of false positives. The powerful amplification mechanism of LAMP, which is beneficial for having highly sensitive assays, also renders it highly susceptible to carry over contamination, where amplified DNA products from previous LAMP reactions become templates for reamplification (Hsieh, Mage, Csordas, Eisenstein, & Soh, 2014; Tomita et al., 2008). Because this assay produces a large amount of DNA, which can easily spread into the open air, and because the protocol requires opening of tubes, these attributes ultimately lead to increased risk of carry‐over contamination and false‐positive results. High temperature, humidity, and inadequate volume of reagents are known risk factors contributing to carry over contamination (Nagai et al., 2016). False‐positive results may also be obtained through primer‐dimer formation, which is more likely to occur when high concentrations of primers are used, as are in a LAMP assay (Meagher, Priye, Light, Huang, & Wang, 2018). Herein, false positives occurred in the template‐free controls during primer optimization. We addressed the issue by (a) thoroughly sterilizing laboratory spaces/equipment, (b) preparing LAMP reactions separately for CHD‐W and CHD‐Z primers using a UV‐sterilized clean bench, (c) preparing LAMP reactions in a place different to where LAMP reactions were incubated and different to where the tubes were opened for adding the staining reagent, and (d) using fresh aliquots or working stocks each time. Following these measures resolved the issue of false‐positive results for subsequent assays for us, but modified protocols do exist for increasing specificity and sensitivity of LAMP assays if the problem persists for others (Ball et al., 2016; Hsieh et al., 2014; Wang, Brewster, Paul, & Tomasula, 2015).

In terms of time and efficiency in the laboratory, the LAMP protocol was completed in less than two and half hours while the standard molecular sexing protocol required twice as long (~5 hr), along with the need for access to laboratory infrastructure. The LAMP protocol can be further simplified by only carrying out two of the three visualization methods, the staining and UV irradiation, which offer immediate observable diagnostic results. Running a gel serves as an additional visual confirmation and was used here for further validation, but it requires more time and supplies to carry out and is not essential for detecting successful LAMP reactions. Furthermore, running a gel at a remote field site would not be possible, so dropping this step lends the LAMP protocol to be more field applicable, unlike the standard PCR‐based molecular sexing protocol, which relies solely on gel electrophoresis for visualizing results.

The key objective of this study was to develop species‐specific LAMP primers that could be used to easily and quickly identify the gender of juvenile blackcaps both in the laboratory and in the wild. While our protocol was developed and carried out in the laboratory, the CHD‐Z and CHD‐W primers designed herein can be easily implemented in a remote field setting (for a field‐based LAMP protocol, please see Centeno‐Cuadros et al., 2017). Having prepared ready‐mixes that can be used and stored at room temperature, a simple DNA extraction protocol that can be run in a single tube per sample, isothermal amplification requiring a single piece of equipment operated from a car, and an immediate visual diagnosis of results, together, allow for a quick and streamlined process of sex determination. However, for field application we strongly recommend preparing primer mixes for each set and vacuum‐drying them, as Centeno‐Cuadros et al., 2017 describe, to preserve their integrity during transportation to the field.

Finally, for studies that are looking to collect birds or samples from one focal gender, the amount of stress on captured birds with the nontarget gender can be reduced by means of rapid in situ sex determination and subsequent release. Or for studies needing to collect a certain number of birds or samples for both sexes, rapid sex determination can be done on the spot. This eliminates the need to take birds or samples back to a laboratory after every collection, employ traditional molecular sexing techniques, and then return to the field to either release birds of the nonfocal gender or to collect more of the underrepresented sex, respectively.

In conclusion, this work adds to the increasing list of available LAMP primers for various avian species and is a new addition within the Passeriformes order.

## CONFLICT OF INTEREST

The authors declare no conflict of interest. 

Permits: Relevant permits include fieldwork in Spain GB‐88/16/EA/FA/AV/GMV and 10/244406.9/17; TRACES reference for transporting birds within the European Union DE01057000121 (MPI Evolutionary Biology as certified institute 010570574697); AZ V242‐62340/2016 (98‐8/16) blood sampling birds; AZ 3104‐4/021/0033 and AZ 1401‐144/153‐5.2.3 allowing us to work in the field, take birds from the wild, and keep them at the MPI Evolutionary Biology under carefully controlled conditions.

## AUTHOR CONTRIBUTIONS

HK and ML: conceived the study; HK: designed the primers; HK and EBS: optimized the primers and tested the samples; HK and ML: wrote the manuscript. All authors contributed critically to the drafts and gave final approval for publication.

## Data Availability

All relevant data for this study are included in and accessible through this manuscript.
